# Early Metacarpal Bone Mineral Density Loss Using Digital X-Ray Radiogrammetry and 3-Tesla Wrist MRI in Established Rheumatoid Arthritis: A Longitudinal One-Year Observational Study

**DOI:** 10.1155/2015/852989

**Published:** 2015-02-17

**Authors:** Anshul Rastogi, Jakob Algulin, Pamela Mangat, Adrian K. P. Lim, Keshthra Satchithananda, Joseph V. Hajnal, Peter C. Taylor

**Affiliations:** ^1^Joint Department of Medical Imaging, Faculty of Medicine, University of Toronto, Mount Sinai Hospital, 600 University Avenue, Toronto, ON, Canada M5G 1X5; ^2^SECTRA Imtec AB, Teknikringen 20, 583 30 Linkoping, Sweden; ^3^Department of Rheumatology, Royal Free Hospital, Pond Street, London NW3 2GQ, UK; ^4^Department of Radiology, Imperial College Healthcare NHS Trust, Charing Cross Hospital, Fulham Palace Road, London W6 8RF, UK; ^5^Department of Radiology, King's College Hospital NHS Foundation Trust, Denmark Hill, London SE5 9RS, UK; ^6^Division of Imaging Sciences, King's College London, The Rayne Institute, 3rd Floor, Lambeth Wing, St. Thomas' Hospital, London SE1 7EH, UK; ^7^Nuffield Department of Orthopaedics, Rheumatology and Musculoskeletal Sciences, University of Oxford, Oxford OX3 7LD, UK

## Abstract

*Objectives*. Early change in rheumatoid arthritis (RA) is characterised by periarticular osteopenia. We investigated the relationship of early metacarpal digital X-ray radiogrammetry bone mineral density (DXR-BMD) change rate (RC-BMD, mg/cm^2^/month) to longitudinal changes in hand and feet radiographic and wrist MRI scores over 1 year.* Materials and Methods*. 10 RA patients completed the study and had wrist 3T-MRI and hand and feet X-rays at various time points over 1 year. MRI was scored by RAMRIS, X-ray was done by van der Heijde modified Sharp scoring, and RC-BMD was analysed using dxr-online.* Results*. There was good correlation amongst the two scorers for MRI measures and ICC for erosions: 0.984, BME: 0.943, and synovitis: 0.657. Strong relationships were observed between RC-BMD at 12-week and 1-year change in wrist marrow oedema (BME) (*r* = 0.78, *P* = 0.035) but not with erosion, synovitis, or radiographic scores.* Conclusion*. Early RC-BMD correlates with 1-year wrist BME change, which is a known predictor of future erosion and joint damage. However, in our pilot study, early RC-BMD did not show relationships to MRI erosion or radiographic changes over 1 year. This may reflect a slower kinetic in the appearance of MRI/radiographic erosions, generating the hypothesis that RC-BMD may be a more sensitive and early structural prognostic marker in RA follow-up.

## 1. Introduction

Radiographic imaging (X-ray) has traditionally been important in diagnosis, as per 1987 American College of Rheumatology (ACR) criteria [1] and subsequent evaluation of patients with rheumatoid arthritis (RA) [2–4]. Evaluation of the extent and rate of structural damage in routine clinical practice involves hand and feet radiographs [5, 6] and the findings may inform treatment change and optimisation.

An early radiographic change in RA is periarticular osteopenia [7]. Early bone mineral density loss is a predictor of differentiation to RA in undifferentiated arthritis [8] and also predicts future joint damage in RA [9]. Plain radiographs have the limitation that they do not assess synovitis and bone marrow oedema (BME) [10]. It is known that BME is an early disease activity measure that predicts future erosions [11].

The time and costs involved in having repeated MRI as a follow-up imaging modality limits its potential. Radiographs are a cheaper, more readily available, quicker, and routinely performed investigation in clinical practice for RA follow-up. Various X-ray scoring methods have been described to assess joint damage in RA in the context of clinical trials [3].

Rosholm et al. described a new automated radiogrammetric method to assess bone mineral density loss from single hand radiographs [12]. This technique has been used in early RA [9, 13, 14]. To date, few studies have compared this method with MRI disease activity change over time [15–17]. In this study, we evaluated the relationship between automated early metacarpal bone mineral density loss, disease activity using high field strength 3T wrist MRI, and hand and feet radiographic scores over a year in patients with established RA undergoing standard clinical care.

## 2. Material and Methods

### 2.1. Patients

The study was approved by local research ethics committee (reference: 06/Q0401/97) and informed written consents were obtained according to the Declaration of Helsinki guidelines. Thirteen rheumatoid arthritis patients, as per 1987 ACR criteria, were enrolled. Ten patients completed study (1 patient withdrew due to claustrophobia, 1 dropped out after baseline and another after week 12).  Table 1 shows completed study group demographics. 1 patient did not attend week 12 visit.

Inclusion criteria included subjects aged ≥18 years, diagnosed with RA as per revised 1987 ACR criteria and had evidence of current or recent active disease with poor prognostic markers for joint damage, as evidenced by rheumatoid factor or anticyclic citrullinated protein antibodies (anti-CCP) positive or at least two radiographic erosions. They were also required to have a swollen joint/s in the hand to be MRI scanned. Exclusion criteria included history of drug or alcohol abuse, MRI contraindications, estimated glomerular filtration rate (eGFR) <60 mL/min, contrast allergy, pregnancy/nursing, blood donation, Steinbrocker function score stage IV, subject unable to position in the scanner, recent hand joint injection, current or recent biological antirheumatic treatment, or any other subject deemed unsuitable by the investigator.

Patients had 5 study visits: day 1, week 4, week 12, week 24, and week 52. At all visits, various clinical assessments were performed, which amongst others included erythrocyte sedimentation rate (ESR), C-reactive protein (CRP), 28-joint disease activity score (DAS28), joint assessments, and MRI safety check including blood test, pregnancy test, and 3T wrist MRI. Hand and feet radiographs were performed at all visits except week 4. Drug history with respect to disease modifying drugs (DMARDS) as part of their standard clinical care was documented and included methotrexate (*n* = 9), sulfasalazine (*n* = 3), hydroxychloroquine (*n* = 3), prednisolone (*n* = 3), and interim depomedrone intramuscular injection for flare up (*n* = 2).

### 2.2. Imaging Protocols

3T wrist MRI (Philips Achieva) was performed using a dedicated SENSE wrist coil in a purpose built subject “bridge” positioning device [18, 19] to allow for similar and comfortable wrist positioning in a longitudinal fashion.

Imaging parameters used were (1) T2w TSE: TR/TE/FA: 9000 ms/55 ms/90°, FOV: 120 × 97 × 82 mm³, acquisition matrix: 208 × 168, slices: 140 (thickness: −0.58 mm, order: interleaved), reconstructed voxel: 0.54 × 0.54 × 1.16 mm, Time: 7 min 48.2 sec; (2) pre- and postcontrast T1wFFE: TR/TE/FA: 11 ms/2.3 ms/20°, FOV: 120 × 98 × 82 mm³, acquisition matrix: 240 × 196, slices: 164 (scan mode: 3D), reconstructed voxel: 0.5 × 0.5 × 0.5 mm, and time: 5 min 55.4 sec; (3) dynamic contrast enhanced (DCE): TR/TE/FA: 3.8 ms/2.1 ms/20°, FOV: 120 × 95 × 80 mm³, acquisition matrix: 96 × 75, slices: 127 (scan mode: 3D, Technique: T1FFE), reconstructed voxel: 1.25 × 1.25 × 0.63 mm, dynamic time: 10.3 sec, and time: 6 min 52.8 sec. There was a 40 sec delay from the start of image acquisition to contrast injection (Gadolinium-DTPA (Gd) (0.2 mL/kg)). A total of 5080 images were acquired (127 slices with 40 frames); (4) T1wFFE proset: TR/TE/FA: 11 ms/3.5 ms/20°, FOV: 120 × 98 × 82 mm³, acquisition matrix: 240 × 196, slices: 164 (scan mode: 3D), reconstructed matrix: 0.5 × 0.5 × 0.5, and time: 5 min 55.4 sec.

### 2.3. Imaging Analysis

Registered and aligned anonymized wrist MRI scans (*n* = 52) were scored using OMERACT RAMRIS [20]. This scoring method scores the wrist joint, using an atlas, for synovitis (0–3) at three points in the joint (maximum score of 9), erosions (0–10) for each bone (maximum score 150), and marrow oedema (0–3) for each bone (maximum score 45). Scoring was done by two experienced (more than 5 years) radiologists (Keshthra Satchithananda and Adrian K. P. Lim). Radiologists were blinded and scored the MRI scans independently in a random order without knowing either the time point or disease status of the subjects, and mean scores were calculated. X-rays in chronological order (*n* = 43) were scored jointly on PACS using van der Heijde modified Sharp (vdH Sharp) scoring, evaluating both hands and feet for erosion and joint space narrowing [3].

**Figure 1:**
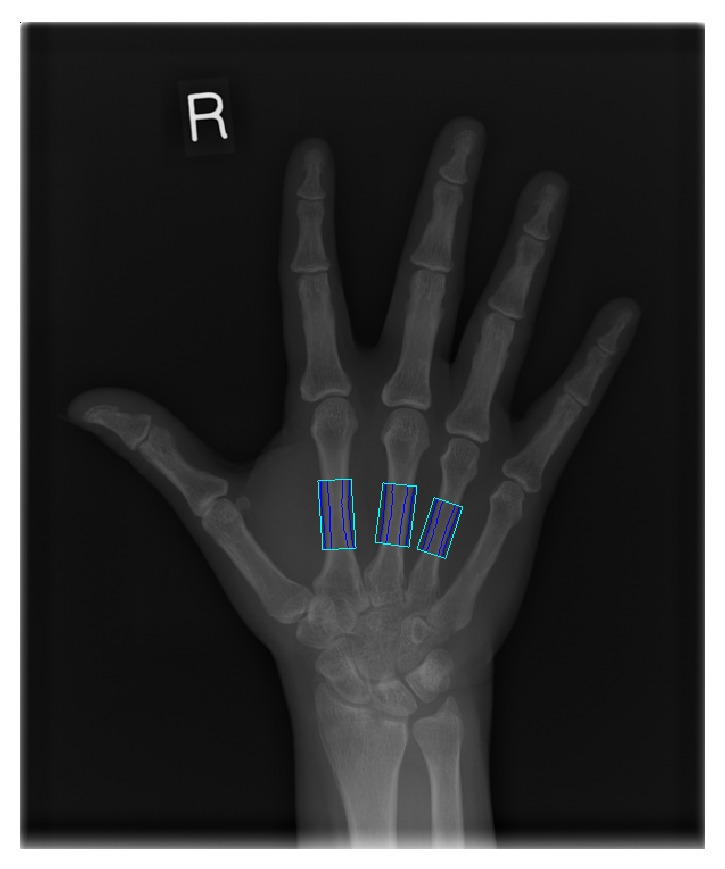
It shows region of interest (ROI) placed on (2–4)th metacarpals. Cortical thickness and bone width are calculated for each point with multiple such measurements made over the ROI, allowing for DXR-BMD to be calculated.

Digital X-ray radiogrammetry (DXR-online, SECTRA, Sweden) was used to calculate DXR-BMD [12]. Using automated algorithms, the computer identifies second to fourth metacarpal diaphysis on digital hand radiographs and places regions of interest (ROI) for a length of 2 cm, 1.8 cm, and 1.6 cm for 2nd, 3rd, and 4th metacarpal, respectively. Cortical thickness and bone width are calculated for each point and multiple such measurements made over the ROI (Figure 1). The final DXR-BMD is calculated based on the formula [12] DXR-BMD = *c*∗VPAmc∗(1 − *P*), where *c* is a constant, *P* is an estimated porosity, and VPA is the weighted average of bone volume per projected area. The rate of change in DXR-BMD (RC-BMD) (mg/cm^2^/month) was assessed. 9 patients' radiographs were analysed over the year and 8 patients' data were available at 12 weeks. 1 subject did not have MRI BME assessment at week 52; hence, only 7 pairs of datasets with week 12 RC-BMD and week 52 MRI were available.

### 2.4. Statistical Analysis

A repeated measure analysis of variance (ANOVA) was performed to evaluate radiographic and MRI scores over time. Normality was tested using Shapiro-Wilk test. Minimal detectable change at 1 year, MDC_95_ (95% confidence), was calculated. For normally distributed data, Pearson correlation was used; otherwise, spearman correlation was used, to evaluate statistical correlation between rate of change in bone mineral density (RC-BMD) and various MRI and X-ray scores. Interclass correlation coefficient (ICC) was used to assess total RAMRIS scores between two readers. A two-way mixed model with consistency type was used. SPSS software was used for analysis. *P* values <0.05 (2 tailed) were considered statistically significant.

## 3. Results

The wrist MRI disease activity scores of this cohort of patients on standard routine treatment over the year remained stable; mean changes (% of maximum score) in synovitis, erosion, and BME scores were −0.7 (−7.7%), 2.4 (1.6%), and 0.4 (0.8%), respectively, at 1 year (Table 2). These were not significant. The minimal detectable change, MDC_95_ (95% confidence), at 1 year for MRI synovitis, erosion^*^, and BME were 3.56, 16.51, 5.97, respectively, with standard error of measurement for synovitis: 1.28, erosion^*^: 5.95, and BME: 2.15 (^*^1 subject with fused bones was excluded).

There was good correlation amongst the two independent blinded scorers for MRI measures and interclass correlation coefficient (ICC) single measures for erosions: 0.984, BME: 0.943, and synovitis: 0.657 (Table 3).

Total hand and feet X-ray scores showed a small increase from baseline score of 33.6 to end of year score of 35.4 (out of a maximum score of 448), thereby an increase of 0.4%. This was not significant. The MDC_95_ for total X-ray score was 4.56 and standard error of measurement was 1.64. 4 out of 10 RA patients showed no change in score at 1 year. 2 patients progressed with hand erosions and increase in score of 1 and 3, respectively. 1 patient had progression in hand and feet by 8 score points, more weighted in the feet (6 score points), which had erosion and joint space narrowing but only slight increase in joint space narrowing in hand with an increase in score by 2. 3 patients progressed in feet alone. No significant differences were seen in radiographic scores over time.

RC-BMD (mg/cm^2^/month) was similar over 12 weeks and 1 year, −0.48 ± 1.5 and −0.55 ± 1.2, respectively (Table 4). 12-week RC-BMD showed no significant correlation with any 12-week and 24-week change scores. 12-week RC-BMD correlated with BME change (*r* = 0.78, *P* = 0.035) and ESR change (*ρ* = 0.91, *P* = 0.001) at 1 year. No correlation was seen with the change in DAS28, MRI erosion, synovitis, or X-ray scores.

**Figure 2:**
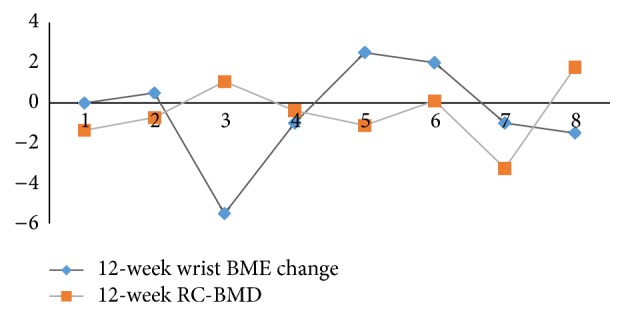
12-week RC-BMD change mapped with 12-week wrist BME change for 8 rheumatoid patients over a year. Majority of patients with low RC-BMD had increased BME change and majority of patients with increase in RC-BMD had reduced BME.

**Figure 3:**
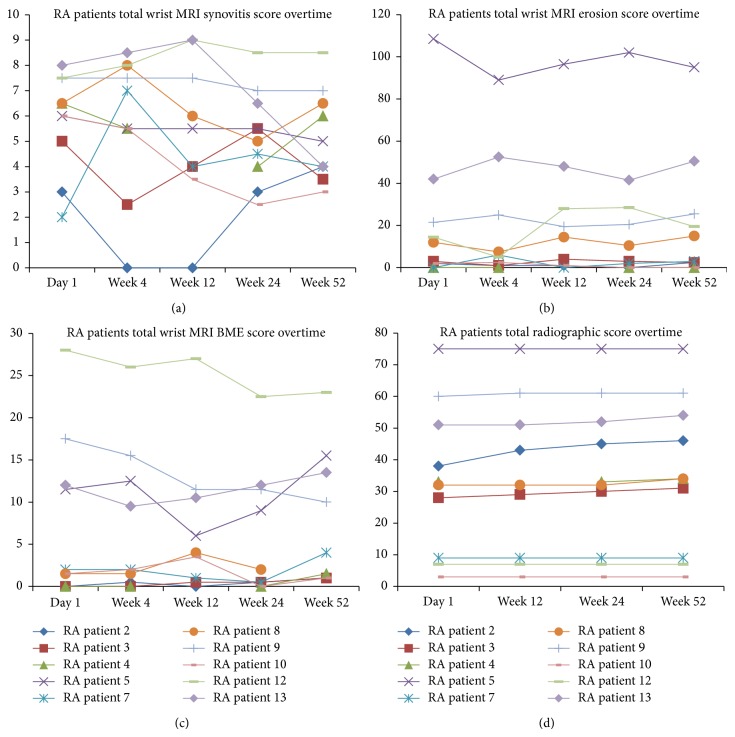
Line graph of MRI synovitis, erosion, BME, and radiographic scores over time for each patient.

Figure 2 shows 12-week RC-BMD and 12-week BME change plotted for each patient.  Figure 3 shows line graph for all patients with MRI and X-ray scores over time.

## 4. Discussion

Plain radiographs form a routine and widely used way of assessing RA joint damage in clinical practice [4, 5]. Application of newer imaging modalities, such as MRI, plays a more crucial role in identifying early changes, like early erosions, synovitis, and BME, that lead to radiographic damage and morbidity on the long term. There has been an increasing trend towards using these in clinical trials with comparison made to changes on radiographs [21–23].

The early changes on radiographs, that is, bone mineral density loss, are quite commonly seen in RA. Loss of metacarpal bone mineral density is known to predict RA development in recent onset arthritis [8]. It is also described as an independent predictor of future damage in RA patients and potentially an important tool in daily clinical work [24].

Inflammatory cytokines such as TNF and IL6 have been linked with increased osteoclast activity, which have been associated with alteration of bone metabolism in early RA [25, 26]. Therapies inhibiting inflammatory cytokines have shown to reduce bone loss in RA [27]. Bone marrow edema in RA indicates the presence of active inflammation and osteitis, which is also associated with inflammatory cytokines [28]. These have shown improvement with anti-TNF treatment [29].

In the present study, we saw strong association between these two measures. There were significant correlations between early (12 weeks) RC-BMD and 1-year change in wrist BME. This could indicate that DXR-BMD change possibly mirrors osteitis seen on MRI macroscopically to already known microscopic and cytokine associations.

Stewart et al. revealed that 1-year change in DXR-BMD in RA patients predicts who will become erosive at 4 years [30]. In early phase clinical trials, early imaging predictive biomarkers are required, and thus DXR-BMD offers potential. In the present study, we evaluated even earlier DXR-BMD change, that is, over 3 months. This correlated with BME at 1 year, which is known to predict future radiographic joint damage in RA [11]. Also of note was that the RC-BMD change over 12 weeks and 1 year was observed to be similar, though with increased loss at 1 year (Table 4). Hence, this early measure could enable clinicians to use a readily available low cost modality to follow up patients. In recent studies, 3-month hand bone loss and baseline MRI findings were reported to predict 1-year MRI erosion in early RA [15] and large 3-month DXR bone loss was seen in patients with MRI erosion progression [17]. We did not see any significant correlation in our cohort between 3-month RC-BMD and 1-year MRI erosion/radiographic scores. There could be several reasons for this; firstly, our patients were mostly the ones with established RA on standard combination disease modifying therapy. Most studies have looked at early RA or undifferentiated arthritis and these cohorts were often selected for poor prognostic factors and thus exhibited a much faster average rate of structural damage. Secondly, in patients with established RA on standard clinical care, these findings could reflect a slower kinetic in the appearance of MRI/radiographic erosions than that of RC-BMD change reflecting more rapid periarticular bone loss and thus generating the hypothesis that RC-BMD may be a sensitive and early structural prognostic marker in RA follow-up. A major limitation of our study was the small number of patients followed up.

**Figure 4:**
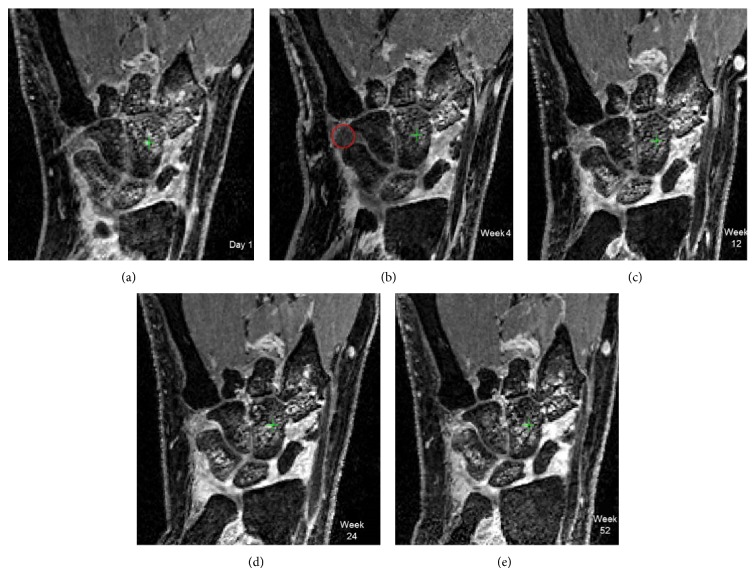
Image shows wrist MRI disease activity at (a) day 1, (b) week 4, (c) week 12, (d) week 24, and (e) week 52 in patient with early disease (disease duration 5 months at baseline). Over the year, there is large amount of BME (RAMRIS scores: 28, 26, 27, 22.5, and 23 at day 1, week 4, week 12, week 24, and week 52). The patient also had rapid loss in RC-BMD at week 12. There is also marked synovitis in the wrist joint with transient improvement at week 4 distal to triquetrum (red circle).

In our small cohort, we observed slight increases in 3 patients (RC-BMD, Figure 2). On further evaluation, it was found that these subjects had a long duration of RA. One patient had fused carpal bones and may have had secondary sclerosis, a finding that could also limit our results. In another subject (disease duration: 5 months), we saw rapid loss in RC-BMD −3.2 mg/cm^2^/month and high BME score throughout the year (day 1, week 4, week 12, week 24, and week 52, with RAMRIS scores: 28, 26, 27, 22.5, and 23 out of 45), Figure 4. Hence, in spite of a small decrease in BME at 1 year, the bone loss continued as the overall burden of osteitis remained large. In future studies, this should be taken into consideration and patients with high osteitis scores included.

It is well known that oral steroids reduce bone mineral density. In our pilot study, of the 8 patients with RC-BMD results at week 12, only 2 patients were on regular oral prednisolone, out of which 1 subject showed increase in RC-BMD. Hence, the results observed in the cohort as a whole for change in RC-BMD over time could not be accounted by the use of steroid therapy.

We used 3T wrist MRI in our study. It is known that at higher field strengths there is better signal to noise ration and hence better resolution [31–34]. This is crucial when imaging small joints, including wrists, which are commonly involved in RA. We also saw good ICC between two independent scorers for the MRI scans.

We noted that stable patients on routine clinical care still have very minimal increase (% of maximum score) in MRI erosion (1.6%) and BME (0.8%) and X-ray radiographic (0.4%) scores. The small increase in X-ray scores was largely due to the progression in feet. Only in 2 out of 10 subjects hand erosion score increased by 1 and 3 score points. This is very small change and would be within the realms of stable disease on visual inspection or measurement error. A reason for the overall stable MRI measures could be mixed cohort of patients in our pilot study, some with early and others with long standing disease with established joint damage. It is possible that the disease kinetics may have a different rate of progression in these subgroups, though this hypothesis will need to be tested in other studies. But even with this small amount of change, we saw correlations between early RC-BMD and 1-year wrist BME, thus offering a promising potential as an early follow-up imaging tool in routine clinical management of RA patients.

Though there are some considerations to be taken into account with DXR analysis, this method requires two images to be acquired 3 months apart and with the same type of X-ray modality. The system automatically rejects change calculations when the same modality type is not used. If all the same types of X-ray modalities were upgraded simultaneously, BMD change during the upgrade period will not be available. The cost of BMD measurements with DXR analysis varies with licensing costs and number of examinations performed, but it is typically, including the cost of the X-ray, less than the cost of MRI.

A limitation of the current pilot study is the small cohort size. These pilot findings generate hypothesis for future studies that will need to be tested in larger, defined patient groups and different MRI imaging platforms. Nevertheless, this is, to our knowledge, the only study to evaluate RC-BMD using DXR and 3T wrist MRI in a longitudinal fashion in established RA patients with wide range of disease duration.

## 5. Conclusions

In conclusion, we have seen in our pilot study that early 12-week RC-BMD change correlates with 1-year wrist BME change. BME is well known to be a predictor of future erosions. However, in this cohort, we did not detect any correlations between early RC-BMD and the progression of radiologic damage in the form of erosions at 1 year. This raises the possibility that in patients with established RA on standardised treatment and a low annualized rate of radiographic progression, DXR may offer a tool as an early indicator of insidious damage progression over the longer term and possibly also of functional loss. Our findings and the generated hypothesis need to be further evaluated in a larger cohort of patients.

## Figures and Tables

**Table 1:** Demographics of completed study group.

	Age (yrs)	BMI	Weight (Kg)	RA duration (months)	ESR (mm/hr)	CRP (mg/L)	TJC/28	SJC/28	Patient global VAS (mm)	DAS28
Mean	53.8	25.9	68.8	68.56	25.3	6.5	5.2	5.7	19.6	3.93
St. dev.	10.6	4.2	10.8	51.5	28.7	3.2	4.0	3.9	12.3	1.3
Range	38–70	19.8–31.8	53–85	5–128	5–100	<2–12	0–12	1–13	1–32	1.54–5.57

BMI: body mass index; ESR: erythrocyte sedimentation rate (mm/hour); CRP: C reactive protein (mg/L); TJC/28: 28 joint count; SJC/28: 28 swollen joint count; DAS 28: disease activity score based on 28 joints.

**Table 2:** Mean and Std. Dev. for MRI disease activity RAMRIS scores over 1 year. The mean total radiographic score change is also shown.

	Baseline	Week 4	Week 12	Week 24	Week 52
Synovitis (0–9)	5.8 ± 1.9	5.8 ± 2.7	5.3 ± 2.9	5.2 ± 1.8	5.1 ± 1.7
Erosions^*^ (0–150)	10.7 ± 13.9	11.1 ± 17.2	14.5 ± 16.9	11.7 ± 15.1	13.1 ± 16.8
BME (0–45)	7.4 ± 9.5	6.9 ± 8.7	7.1 ± 8.5	5.8 ± 7.6	7.8 ± 8.0
Total X-ray score (max 448)	33.6	—	34.4	34.7	35.4

^*^Erosions score excluded a patient with complete fused carpal joints.

**Table 3:** Interreader correlation for RAMRIS scoring.

MRI disease activity	Interclass correlation coefficient (ICC)	95% confidence interval		*n*
Synovitis	0.657	0.46	0.78	51
Erosions	0.984	0.97	0.99	51
BME	0.943	0.9	0.96	50

**Table 4:** DXR-BMD average and rate change (RC-BMD) over time.

	RC-BMD over 12 weeks [mg/cm^2^/month]	Average BMD change over 12 weeks [g/cm^2^]	RC-BMD over 1 year [mg/cm^2^/month]	Average BMD change over 1 year [g/cm^2^]
Average	−0.48	−0.0008	−0.55	−0.006
Std. Dev.	1.5	0.004	1.2	0.01
